# Evidence of the American *Myxobolus dechtiari* was introduced along with its host *Lepomis gibbosus* in Europe: Molecular and histological data

**DOI:** 10.1016/j.ijppaw.2021.04.005

**Published:** 2021-04-15

**Authors:** U. Goswami, K. Molnár, G. Cech, J.C. Eiras, P.K. Bandyopadhyay, S. Ghosh, I. Czeglédi, C. Székely

**Affiliations:** a Institute for Veterinary Medical Research, Centre for Agricultural Research, Budapest, Hungary; b Departamento de Biologia, Faculdade de Ciências, Universidade do Porto, Porto, Portugal; c Balaton Limnological Institute, Centre for Ecological Research, Klebelsberg Kuno u. 3, 8237, Tihany, Hungary; d University of Kalyani, West Bengal, India; e Centro Interdisciplinar de Investigação Marinha e Ambiental (*CIIMAR/CIMAR*), Terminal de Cruzeiros do Porto de Leixões, Matosinhos, Portugal

**Keywords:** *Lepomis gibbosus*, *Myxobolus dechtiari*, Occurrence, Histology, ssrDNA

## Abstract

The American pumpkinseed *Lepomis gibbosus*, was introduced to Europe more than one hundred years ago. Currently it is a common fish in European freshwaters but relatively few specific parasites infect this fish in this new habitat. In Europe only a single species, *Myxobolus dechtiari* seems to represent the American myxosporean fauna of centrarchid fishes. *M. dechtiari* was found in both Portugal and Hungary. This species forms plasmodia with elongated shape inside the cartilaginous rays of gill filaments. In the advanced stage of infection, after disruption of plasmodia, small groups of myxospores remain enclosed in the cartilaginous gill rays causing distortions in the filaments. Myxospores were ellipsoidal in frontal view and lemon-shape in sutural, length 12.5 ± 0.46 (12–13.4) μm, width 10 ± 0.37 (9.6–10.4) μm, and thickness 7.4 ± 0.37 (7–8) μm; the polar capsules were pyriform, equal in size, length 5.6 ± 0.21 (5.3–6) μm, width 3.2 ± 0.16 (3–3.6) μm; Seven to eight polar tube coils were arranged perpendicularly to the capsule length. There was a small, round, 0.4 ± 0.1 (0.3–05) (N = 50) intercapsular appendix in the spores. The small subunit ribosomal DNA (ssrDNA) of *M. dechtiari* differed from other myxozoans sequenced to date. Phylogenetic analysis of the ssrDNA gene sequence placed this species in a clade including actinospores and *Myxobolus* species: Raabeia type1, Triactinomyxon sp., and *Myxobolus osburni* infecting the same host fish.

The focus of our study was to prove that the pumpkinseed, a fish originated from North-America introduced one of its myxosporean parasite to Europe. Emphasis was put on to demonstrate the unique feature of this parasite causing infection in the cartilaginous gill rays.

## Highlights

• American pumpkinseed fish were introduced to Europe as an ornamental fish.
• Relatively few specific parasites are reported from the pumpkinseed in Europe.
• The myxosporean *Myxobolus dechtiari* described originally in Canada infects the introduced host both in Hungary and Portugal.
• SsrDNA sequences of *M. dechtiari* from pumpkinseed fish is provided.

## Graphical abstract

**Figure:**
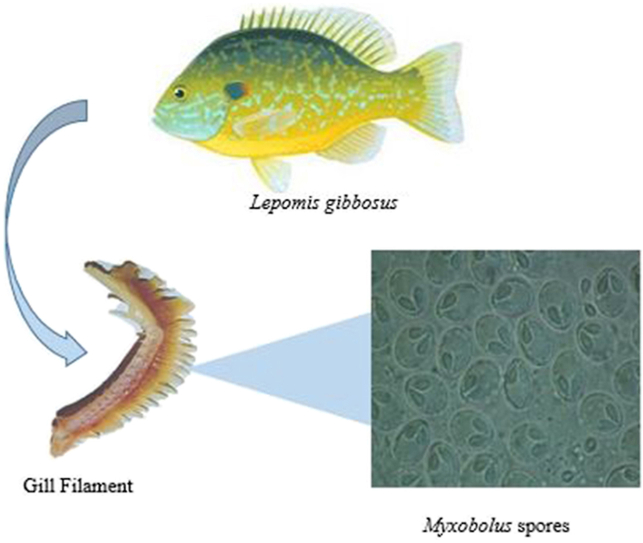

## 1 Introduction

The American pumpkinseed sunfish, *Lepomis gibbosus* Linnaeus, 1758, a common centrarchid fish in North America, was introduced in Europe more than one hundred years ago and became a common member of the fish fauna of several European countries, among them in Hungary and Portugal. The fish brought only some of its parasites to the new habitats but became host of some European parasite species, too (Ondračková et al., 2015). Out of the parasites with American origin, two monogeneans, *Onchocleidus similis* (Mueller, 1936) and *Haplocleidus dispar* (Mueller, 1936) are the best known. These monogeneans were found in Romania by Roman-Chiriac (1960), in Hungary by Molnár (1968), and in the Czech Republic by Ondračková et al. (2011). Out of the helminth parasites of Europe, *Schulmanela petrushewskii* (Shulman, 1948) seems to be a common nematode parasite of pumpkinseed (Shulman, 1948). Kosuthova et al. (2009) described *Proteocephalus percae* (Müller, 1780) and the larva of *Triaenophorus nodulosus* (Pallas, 1781) from pumpkinseed in Slovakia whereas Moshu (2012) reported a series of wide host-range protozoan parasites from Moldova. Recently the unexpected European emergence of metacercariae of *Posthodiplostomum centrarchi* Hoffman (1958) was detected by several authors (Kvach et al., 2017; Stoyanov et al., 2017; Cech et al., 2020).

Myxosporean fauna of pumpkinseeds in America is relatively well studied. Nine *Myxobolus* species have been described from this species so far (Cone & Anderson 1977a, 1977b; Li and Desser 1985). Cone (2001) revised the validity of *Myxobolus* spp. from the pumpkinseed and confirmed 6 species infecting *L. gibbosus*, namely *M. dechtiari* Cone and Anderson, 1977, *M. gibbosus* Herrick, 1941*, M. magnaspherus* Cone and Anderson, 1977, *M. osburni* Herrick, 1936, *M. paralintoni* Li and Desser (1985) and *M. uvuliferis* Cone and Anderson, 1977. Out of the above species only *M. dechtiari, M. gibbosus* and *M. uvuliferis* are known to infect the gills. The first *Myxobolus* infection on the gills of *L. gibbosus* in Europe was found by Lom (1969), who identified it as *Myxobolus* cf. *carelicus* Perushewsky, 1940. Moshu (2012) who studied the protozoan fauna of pumpkinseed in Moldova found also this *Myxobolus* species and identified it as *M. dechtiari* Cone and Anderson, 1977**.**

In the present paper, the authors report on the occurrence of the North-American *Myxobolus dechtiari* from pumpkinseed *Lepomis gibbosus* in rivers of Portugal and Hungary, pointing out that the species was introduced and established in the continent along with its host. This species forms plasmodia in the cartilaginous gill rays of gill filaments and groups of myxospores enclosed by cartilage cells could be found in chronic cases.

## 2 Materials and methods

Seven specimens of pumpkinseeds *L. gibbosus* (L.), 7–9 cm long, were collected by an electric fishery device from the Neiva River (41° 36′ 57.4″ N, and 8° 45′ 30.5″ W), close to Porto, Portugal in 2008. Myxospores found in gill cartilage of infected fishes were photographed. Another part of myxospores were fixed in 10% formalin for histological purpose, while the rest was preserved in 80% ethanol for molecular studies. All further studies on this material were executed in Hungary. Pumpkinseed specimens of 5–16 cm long were seined from the Lake Balaton, from the Danube River, from the Egervíz Creek north of Lake Balaton and the Sió canal during regular fish health surveys since 2009 until 2015 in Hungary. Intensified investigation of pumpkinseeds started in 2015 when a freshly introduced trematode metacercaria was recorded in pumpkinseed specimens in a water reservoir in North Hungary (Cech et al., 2020). In 2018, besides seining at different sites of Lake Balaton: Tihany (46° 54′ 51.6″ N, 17° 53′ 36.5″ E), Balatonvilágos (46° 58′ 0″ N, 18° 9′ 27″ E), Siófok (46° 54′ 46″ N, 18° 2′ 34.6″ E), Balatonszemes (46° 48′ 9″ N, 17° 44′ 41.5″ E), Keszthely (46° 45′ 22.1″ N, 17° 14′ 59.6″ E) fish were also collected by electric fishing. In Hungary, the gills of 129 pumpkinseed specimens with length of 5–16 cm in total were examined for myxosporean infections. Out of them, 2 specimens were collected from the Egervíz Creek (46° 47’ 26.6″ N, 17° 27′ 50.5″ E), 7 specimens from a North Hungarian Water reservoir (47° 59′ 44.9″ N, 19° 51′ 13.1″ E), 2 from the Danube River (47° 40′ 55.6″ N, 19° 4′ 53.7″ E) and 98 fish from the Lake Balaton and 20 specimens derived from the Sió channel close to the draining ditch built between the lake and the channel.

Fish were carried to the laboratory alive, in oxygenated plastic bags, kept in aerated aquaria and subjected to complete parasitological dissection mostly within three days. When mature plasmodia were found, some of the myxospores were studied in fresh preparations, some of them were collected into Eppendorf tubes and stored at −20 °C until further molecular use, while the rest of the myxospores were preserved in glycerine-gelatine as slide preparations or used for transmission experiments. Tissue samples of infected organs containing developing and mature plasmodia were fixed in Bouin's solution, and after dehydrated in ascending series of ethanol and acetone, they were embedded in paraffin wax, cut to 4–5 μm sections, and stained with hematoxylin and eosin. The vitality of myxospores was checked by adding myxospores to 0.4% of solution of urea.

Myxospores of a given plasmodium were regarded as matured when at least 90% of the myxospores extruded polar tubes in this solution. Unfixed myxospores were studied by Nomarski differential interference contrast of an Olympus BH2 microscope. The myxospores were photographed with an Olympus DP10 digital camera or recorded on videotapes, and digitized images were obtained. All measurements are given in μm. Measurements of some fresh myxospores were taken with a calibrated eyepiece micrometer (μm) according to the guidelines of Lom and Arthur (1989) while the majority of myxospores were measured from the digital images. Molecular data were obtained from myxospores collected in Hungary. Molecular data were obtained from myxospores collected in Hungary.

### 2.1 Genomic DNA isolation and sequencing

Preserved isolated plasmodia or myxospores in 95% ethanol were centrifuged at 8000×*g* for 10 min. Genomic DNA was isolated from the obtained pellet using the Genaid Tissue Genomic DNA Mini kit, following the manufacturer's recommended protocol for animal tissue. ssrDNA was amplified by semi-nested PCR: with a first round using universal primers ERIB1 and ERIB10 (Barta et al., 1997). The reaction mixture consisted of 14.4 μl nuclease-free water, 2.5 μl of 10× DreamTaq buffer (Thermo Scientific, Vilnius, Lithuania), 0.1 μl of DreamTaq Polymerase (1 U; Thermo Scientific), 0.2 mM dNTPs (Thermo Scientific), 0.325 μM of each primer and 2 μl of the extracted DNA in a final volume of 25 μl. The following profile was used for amplification: an initial denaturation step at 95 °C for 3 min followed by 40 cycle at 95 °C for 1 min, 55 °C for 1 min, 72 °C for 2 min and completed with terminal extension step at 72 °C for 7 min. This was followed by two semi-nested PCR reactions that generated over-lapping sequences, using ERIB1 with CR1R (Székely et al., 2015) and ERIB10 with CR1F (Székely et al., 2015). The reaction mixture contained 31.8 μl nuclease-free water, 5 μl of 10× DreamTaq buffer (Thermo Scientific), 0.2 μl of DreamTaq polymerase (2 U; Thermo Scientific), 0.2 mM dNTPs (Thermo Scientific), 0.325 μM of each primer and 1 μl from the first round PCR product in a final volume of 50 μl. The amplification conditions were: 95 °C for 3 min, followed by 35 cycles at 95 °C for 50s, 55 °C for 50s, 72 °C for 1 min 40s, and terminated with an extension step at 72 °C for 7 min. The primer sequences are listed in Table 2. Amplified products were analyzed by electrophoresis in a 1% agarose gel. The PCR products were resected from the gel, purified with the Gel/PCR DNA Fragments Extraction Kit (Geneaid Tawai city, Taiwan) and directly sequenced with sequencing primers (Table 2) in both directions using the BigDye Terminator v3.1 Cycle Sequencing Kit (Life Technologies) with an ABI PRISM 3100 Genetic Analyser (Life Technologies), using the amplification and inner primers.

**Table 1:** Spores measurements of *Myxobolus dechtiari* found in Portugal and Hungary in Pumpkinseed Fish.

	*Myxobolus dechtiari.* from Portugal	*Myxobolus dechtiari* from Hungary	*Myxobolus dechtiari* by Cone and Anderson, 1977a, Cone and Anderson, 1977b
Location of plasmodia	Cartilaginous gill rays	Cartilaginous gill rays	Basal cells of gill lamellae
Shape and size of plasmodia	Round or short ellipsoidal	elongated	elongate
Spore shape in frontal view	Ellipsoidal, somewhat oval	Ellipsoidal, somewhat oval	ovoid
Spore length	12 ± 0.21 (11.5–12.3)	12.5 ± 0.46 (12–13.4)	11.5 (10–14)
Spore width	9.5 ± 0.22 (9–10)	10 ± 0.37 (9.6–10.4)	8 (7–9)
Spore thickness	–	7.4 ± 0.37 (7–8)	7.5 (7-8
Length of polar capsules	5.4 ± 0.18 (5.1–5.6)	5.6 ± 0.21 (5.3–6)	4–6
Width of polar capsules	3.1 ± 0.14 (2.9–3.4)	3.2 ± 0.16 (3–3.6)	2–3
Intercapsular Process length	0.4–0.5	0.5–0.7	Not found
Number of polar filament coils	7–8	7–8	7–8

**Table 2:** Primers used in PCR and sequencing.

Primers	Sequences	References
ERIB1	ACCTGGTTGATCCTGCA	Barta et al. (1997)
ERB10	CTTCCGCAGGTTCACCTACGG	Barta et al. (1997)
CR1R	GAT YAG ATA CCG TCS TAGT	Székely et al. (2015)
CR1F	CGA AGA CGA TCA GAT ACC GTC CTA	Székely et al. (2015)
MYXGEN4F	GTGCCTTGAATAAATCAGAG	Diamant et al. (2004)
MB3	GATGATTAACAGCAGCGGTTG	Molnár et al. (2002)
MB5	ACCGCTCCTGTTAATCATCAC	Molnár et al. (2002)

### 2.2 Phylogenetic analysis

Obtained sequence fragments were assembled by using MEGA X (Kumar et al., 2018) and manual adjustments were performed to correct or remove ambiguous positions from the dataset. Assembled ssrDNA sequences were verified as myxozoan by GenBank BLAST search and related Myxozoan sequences were added to the alignment. The selected sequences were restricted to only those of at least 1000 base pairs in length and 87% similarity. The sequences were aligned by using Clustal W (Thompson et al., 1994) implemented in the MEGA X. Pairwise distances were computed by using the p-distance model matrix in MEGA X. The evolutionary history was inferred by using the Maximum Likelihood method and General Time Reversible model with a bootstrap of 1000 replicates. The best evolutionary model of nucleotide substitution using the Akaike Information Criterion (AIC) was determined with the MEGA X. The tree with the highest log likelihood (−10261.14) is shown. The percentage of trees in which the associated taxa clustered together is shown next to the branches. Initial tree(s) for the heuristic search were obtained automatically by applying the Maximum Parsimony method. A discrete Gamma distribution was used to model evolutionary rate differences among sites [5 categories (+*G*, parameter = 0.4039)]. The tree is drawn to scale, with branch lengths measured in the number of substitutions per site. This analysis involved 23 nucleotide sequences. All positions with less than 95% site coverage were eliminated, i.e., fewer than 5% alignment gaps, missing data, and ambiguous bases were allowed at any position (partial deletion option). There were a total of 1195 positions in the final dataset. *Chloromyxum cristatum* (AY604198) was chosen as an out-group in the final alignment.

## 3 Results

**Fig. 1:**
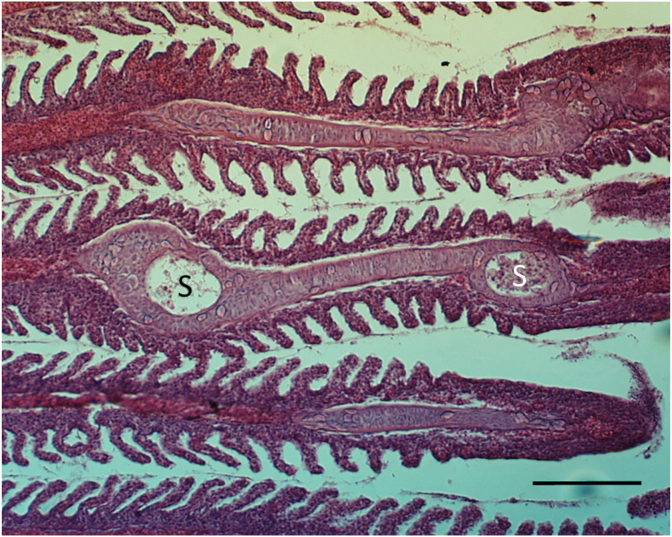
Group of spores (S) of *M. dechtiari* enclosed into the distorted cartilaginous gill ray in a pumpkinseed from Portugal. H. & E., Bar = 100 μm.

An intensive invasion of the gill cartilage by mature *Myxobolus* myxospores were found in three of the examined pumpkinseeds from Neiva River in Portugal at the end of June 2008 (Fig. 1). Plasmodia delimitations were not observed, suggesting aged plasmodia.

**Fig. 2:**
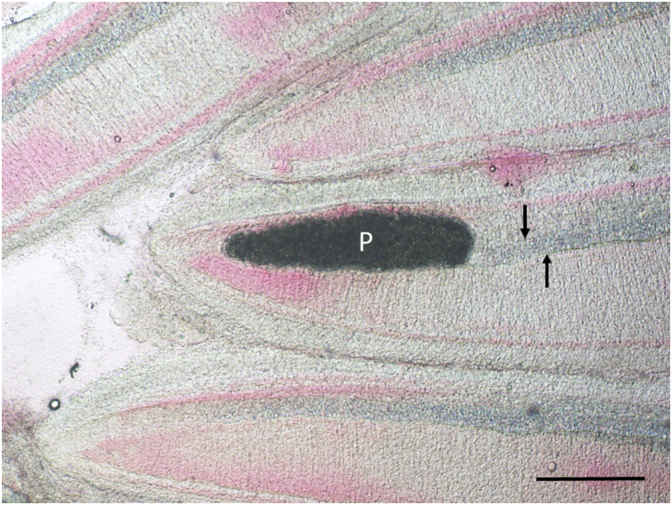
Gill filaments of a pumpkinseed collected in Hungary. Close to the tip of the filament a *Myxobolus dechtiari* plasmodium (P) in continuation of the cartilaginous gill ray (arrows) is located. Fresh mount. H. & E., Bar = 100 μm.

**Fig. 3:**
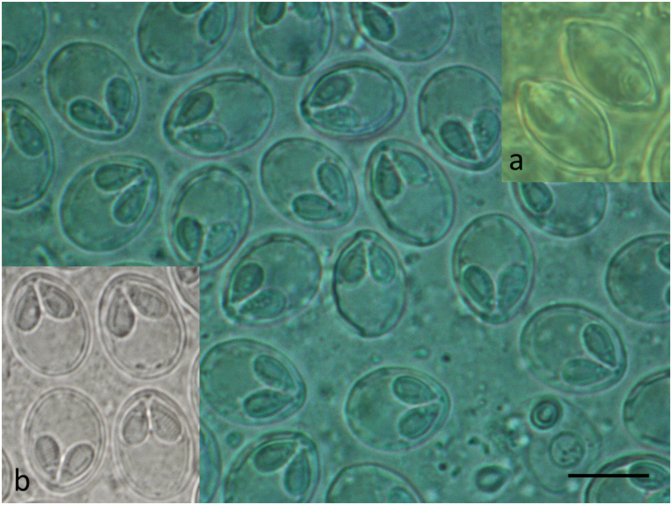
Myxospores of *M. dechtiari* collected in Hungary in frontal view. Inset a: myxospores of *M. dechtiari* in sutural view. Inset b: Myxospores in frontal view collected in Portugal. Fresh mount. Bar = 10 μm.

Myxospores obtained from the cartilage were only photographed (Fig. 3b inset) and measured. Matured plasmodia in these fishes were not observed. Myxospores (Table 1) obtained from cartilage corresponded to the shape and size of the spores found in Hungary and to those which were described by Cone and Anderson, 1977a, Cone and Anderson, 1977b. Following the detection of *M. dechtiari* in Portugal, pumpkinseeds was more carefully studied also in Hungary, but only a single specimen proved to be infected in July 2009. This specimen, which was collected from the Egervíz Creek, was infected with plasmodia containing matured myxospores. Further plasmodia were found only in three fishes of the examined 20 specimens collected from the Sió channel in 2018. Plasmodia were located at the tips of the gill filaments (Fig. 2). 20 to 44 plasmodia were counted in the infected specimens. At the site of plasmodial development, gill filaments became distorted. Myxospores from the Portuguese and Hungarian cases were similar both in their shape and in size (Table 1). Myxospores found in this study corresponded in shape and size to the original description of the species *Myxobolus dechtiari*.

### 3.1 Morphological characterization of *Myxobolus dechtiari*Cone and Anderson, 1977a, Cone and Anderson, 1977b

**Fig. 4:**
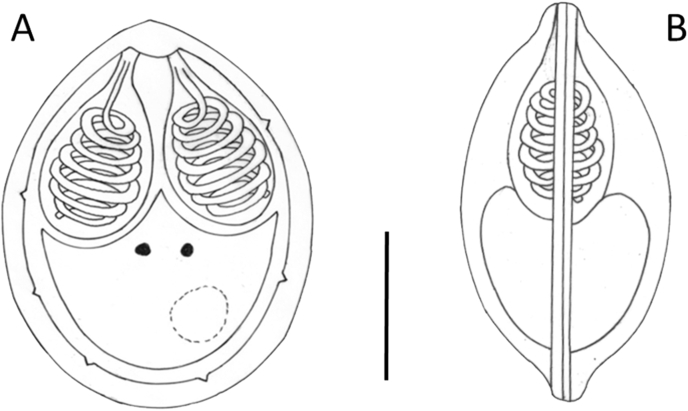
Schematic drawings of *M. dechtiari* myxospores. A) Spore in frontal view, B) Spore in sutural view. H. & E., Bar = 10 μm.

Matured plasmodia elongated in shape measuring 1.5–2 mm in length and 0.5–0.7 mm in diameter located typically at the distal regions of gill filaments (Fig. 2). Myxospores ellipsoidal in frontal view (Fig. 3, Fig. 4) and lemon-shape in sutural view (Fig. 3a inset) measured 12.5 ± 0.46 (12–13.4) (N = 50) in length, 10 ± 0.37 (9.6–10.4) (N = 50) in width, and 7.4 ± 0.37 (7–8) (N = 10) in thickness. Polar capsules pyriform, equal in size, slightly converging anteriorly, 5.6 *±* 0.21 (5.3–6) (N = 50) in length, 3.2 *±* 0.16 (3–3.6) (N = 50) in width (Table 1). Seven to eight polar tube coils arranged perpendicularly to the capsule length wounded densely in the polar capsule. A small, round, 0.4 ± 0.1 (0.3–0.5) (N = 50) intercapsular appendix present in the myxospores. Sutural protrusion forming a circular rim around the spore emerging about 0.7 over the surface of the spore (Fig. 3a inset). In sutural view both at the anterior and posterior end a 0.9 (0.8–1), protrusion seen. The thickness of the rim in sutural view measured about 0.5–0.7. The fresh myxospores showed 5 to 7 clear sutural edge markings. A single binucleated sporoplasm with an iodinophilous vacuole present. Mucous envelope absent.

Myxospores preserved in 70% ethanol and photo types were deposited in the parasitological collection of the Zoological Department, Hungarian Natural History Museum, Budapest, Coll. No. HNHM-PAR-20891. The ssrDNA sequences of *M. dechtiari* were deposited in the GenBank under the accession number MW588907 and MW588908.

**Host:** Pumpkinseed *Lepomis gibbosus* (L.) (Centrarchidae).

**Locality:** Sió channel, Hungary, City Siófok, (46° 54′ 46.3″ N, 17° 53′ 16.8′ E)

**Additional locality:** Neiva River, Portugal and Egervíz Creek, Hungary.

**Site of tissue development:** Cartilaginous gill rays of filaments.

**Molecular and phylogenetic analyses:** Unfortunately, due to fixation problems obtaining DNA sequences from the myxospore samples of Portugal did not succeed. However, two isolates of *M. dechtiari* collected in Hungary were sequenced (ssrDNA) and evaluated in phylogenetic analysis. These two sequences had genetic similarity of 99.6%, and the BLASTn search in the NCBI database of did not match any known species with a similarity above than 95%. The closest similarities were observed with two actinospores samples, Raabeia sp. type 1 (KJ152184, 94.2–94.8%) from the oligochaete host *Isochaetides michaelseni* and Triactinomyxon sp. (AF378351, 91.3–91.8%) from *Limnodrilus hoffmeisteri* and *Myxobolus osburni* (AF378338, 91.1–91.3%) from the same host fish. Sequences of *M. dechtiari* was placed in a single branch with maximum bootstrap support on the phylogenetic tree. They were also located in a larger monophyletic clade including the above-mentioned three myxozoan samples also with maximum bootstrap support. Other genetically related myxozoan sequences were placed in different phylogenetic lineages often with high bootstrap values (Fig. 9).

**Fig. 5:**
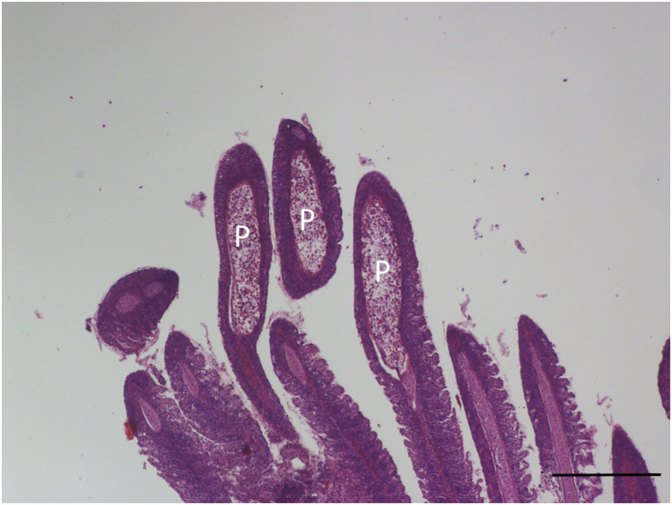
*M. dechtiari* plasmodia (P) in the tip of gill filaments of the pumpkinseed. H. & E., Bar = 200 μm.

**Fig. 6:**
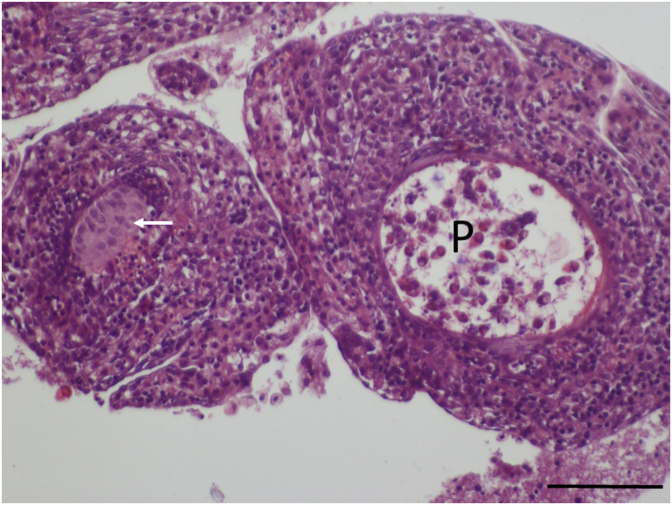
*M. dechtiari* plasmodium (P) in a cross-section of gill filament. In the neighboring uninfected filament the cartilaginous gill ray (arrow) is well seen. H. & E., Bar = 100 μm.

**Fig. 7:**
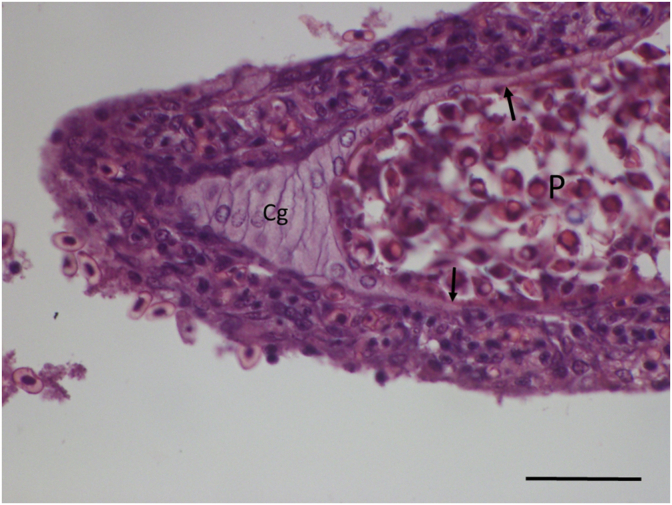
Posterior end of *M. dechtiari* plasmodium (P) close to the tip of filament. The gradually thinning gill ray surrounds the plasmodium (arrows). H. & E., Bar = 50 μm.

**Fig. 8:**
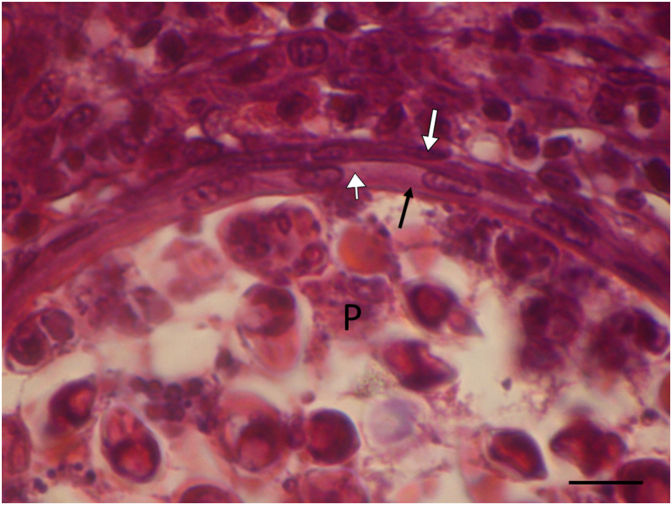
Cross-section of *M. dechtiari* plasmodium (P) at its border region. Between the double membrane of the plasmodial wall some chondrocyte are seen (arrow) H. & E., Bar = 100 μm.

**Histology:** Elongated shape plasmodia filled with myxospores (Fig. 5) located in the gill filaments as a continuation of the cartilaginous gill arches. At this part of the filament, the cartilaginous gill ray was not observable. In a similar way at low magnifications no cartilaginous elements were seen around plasmodia cross-sectioned at the central part of the plasmodia, though the cartilage of the gill rays of the uninfected filaments was clearly seen (Fig. 6). At this part, the plasmodium was surrounded by a double contoured thick eosinophil capsule. At its anterior and posterior end, the plasmodium joined to the cartilaginous gill ray so that in a short part the gradually thinning cartilage cells surrounded the plasmodium (Fig. 7). Cross-section at the central part of the plasmodium showed that the cartilage sheet around the cyst is continuous, and at larger magnification of the attenuate plasmodium wall, a three-layered structure was recognized. In this section covering the ectoplasm of the plasmodium, the flattened nuclei of cartilage cells were well observed (Fig. 8), while the outer wall was composed of the connective tissue sheet of the cartilaginous gill ray, indicating that only the inner wall belongs to the plasmodium. In advanced cases, when plasmodia were not present anymore, small groupss of myxospores enclosed in the cartilaginous gill rays were observed. Gill rays around the enclosed spores thickened and became distorted. At these infected parts, gill lamellae became shorter (Fig. 1). No sign of other host reaction was recorded. The myxospores were separated from the chondrocytes only by a thin eosinophil ectoplasm.

**Fig. 9:**
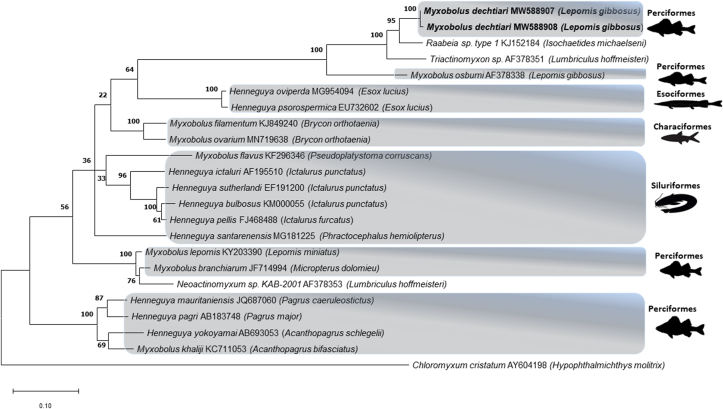
Phylogenetic tree generated by maximum likelihood analysis of ssrDNA sequences of *Myxobolus dechtiari* and other closely related myxosporean species identified by BLAST; GenBank accession numbers and their host name shown after the species name. Numbers at nodes indicate the bootstrap confidence values (ML). *Chloromyxum cristatum* was used as an outgroup.

## 4 Discussion

Planned or accidental introduction of fishes to another continent also often results in carrying some of their parasites to the new habitat (Bauer and Hoffman, 1976). The best example for this possibility is the introduction of *M. cerebralis* with the brown trout to North America (Hoffman, 1970). *M. dechtiari* might have arrived to Europe in a similar way. While *M. cerebralis* in America successfully parasitized new hosts and became an important pathogen in the new habitat, *M. dechtiari* remained only the parasite of the pumpkinseed, and it seems to be the single myxosporean species of this host, which arrived to Europe. The common presence of this species in pumpkinseeds was observed in Portugal, therefore the occurrence of this species in pumpkinseeds from Hungarian natural habitats was expected, first of all from the Lake Balaton where the pumpkinseed is an abundant fish. Dissections made on 158 specimens of this fish from Lake Balaton, however resulted in a negative outcome, *M. dechtiari* could be found only in pumpkinseeds (4 fish out of 22) collected from the inflowing and outflowing tributaries of Lake Balaton. This observation seems to suggest that *M. dechtiari* prefers flowing waters.

The morphological characteristics of the myxospores corresponded to the original description of the species by Cone and Anderson (1977a), except the small intercapsular appendix. Lom (1969) reported on the presence of this small intercapsular appendix in the myxospores, but this feature is not mentioned either in the description by Cone and Anderson (1977a) or later in the review of myxosporeans of pumpkinseed by Cone (2001).

*M. dechtiari* is characterized by its specific histotropism to cartilage, especially to the cartilaginous gill rays. Presumably, Cone and Anderson (1977b) noticed a similar chondrophil location when they stated that plasmodia were among the basal cells at the distal ends of gill lamellae. The latter location seems to be more common in fish, but so far relatively few studies addressed this problem. Molnár et al. (2008) found that *M. feisti* in roach (*Rutilus rutilus* (L)) and *M. susanlimae* in bleak (*Alburnus alburnus* (L.)) cause a similar type of infection. Histological studies made on the plasmodia of *M. dechtiari* seem to prove that this species develops in the cartilaginous gill rays of the filaments. For a closer identification of the location of *Myxobolus* spp. developing in the gill filaments Molnár et al. (2018) suggested the preparation of cross-sections of plasmodia. In the case of preparing only longitudinal sections of the gill filament, it may be supposed that plasmodia obscure only the cartilage; however cross-sectioned preparations can make visible the position of the plasmodia to the cartilaginous gill rays. Cross-sections elucidated that the host origin thick, double wall of the plasmodia is composed of two separate layers, by an inner cartilaginous layer above the thin ectoplasm of the plasmodium and an outer layer composed of stratified connective tissue. Inside the attenuated cartilage layer, less frequently the flattened cartilage cells are also recognizable. In our present case, both longitudinal and cross-sections might indicate that the whole development took place inside the cartilage. However, some data seem to contradict this observation, like in the case of *M. feisti* and *M. susanlimae* spp. myxospores enclosed in the filament cartilage were found commonly (Molnár et al., 2008). Later in a detailed study on young plasmodial stages, Molnár et al. (2012) concluded that *M. feisti* starts its development close to the cartilage and myxospores are enclosed by cartilage cells only by a secondary reaction. In the present case, no studies were performed on early stages, therefore we are not able to give a definite answer whether plasmodia of *M. dechtiari* start their development among cartilage cells or they are engulfed by cartilage in a secondary reaction. Molnár (1994) stated that *Myxobolus* species have a relatively strict host-, organ- and tissue specificity. Besides the morphology of myxospores, these characteristics provide immense help in species identification. Most species develop in a specific organ of the fish; therefore, tissue tropism is one of the most decisive factors for differentiating species with morphologically similar myxospores.

The phylogenetic analysis revealed that the sequence of *M. dechtiari* differs from other species available in the GenBank. This observation is consistent with the general tendency of myxosporean parasites to diverge according to the fish host and the location (Ferguson et al., 2008; Naldoni et al., 2011; Adriano et al., 2012; Carriero et al., 2013; Molnár and Eszterbauer 2015). Consequently, *M. dechtiari* shows the highest similarity to the North-American *M. osburni* described from the same fish species. *M. dechtiari* showed also a somewhat close relationship to a triactinomyxon type actinospore from North-America and a raabeia type from Hungary. This result does not provide any support to decide which type of actinospore could be a possible developmental stage for *M. dechtiari*; *Myxobolus* species have most commonly triactinomyxons as actinospore, however other morphotypes could also exist, like raabeia (Yokoyama et al., 1995; Molnár et al., 1999; Eszterbauer et al., 2006) and echinoactinomyxon (Marton and Eszterbauer 2011). According to the Andree et al. (1999), molecular data (preferably ssrDNA) are required for description, comparison, and identification of Myxozoan species, but our results could not be compared to most of the myxozoan species from pumpkinseed (only to *M. osburni*) due to the lack of sequence data in the Genbank. However, based on the available morphological and molecular data, it is postulated that the myxozoan species found in the Hungarian and Portugal pumpkinseed specimens belong to the species *M. dechtiari*.
